# Expression of vascular endothelial growth factor and matrix metalloproteinase-9 in *Apis mellifera* Lawang propolis extract gel-treated traumatic ulcers in diabetic rats

**DOI:** 10.14202/vetworld.2018.304-309

**Published:** 2018-03-14

**Authors:** Diah Savitri Ernawati, Ade Puspa

**Affiliations:** Department of Oral Medicine, Faculty of Dental Medicine, Universitas Airlangga, Surabaya, Indonesia

**Keywords:** diabetes mellitus, matrix metalloproteinase-9, ulcer healing process, vascular endothelial growth factor expression

## Abstract

**Aim:**

The aim of this study was to determine the effect of *Apis mellifera* propolis extract gel on vascular endothelial growth factor (VEGF) and matrix metalloproteinase-9 (MMP-9) expression in the traumatic ulcers of rats afflicted with diabetes mellitus (DM).

**Materials and Methods:**

The study was conducted on 24 male Wistar rats (*Rattus norvegicus*) induced with DM by injecting 50 mg/kg of Streptozotocin, intraperitoneally, and a traumatic ulcer on their lower lip mucosa. These were divided into eight groups: Four each for control and treatment groups. Each control and treatment group consisted of three rats. The control groups treated with hydroxypropyl methylcellulose 5% gel and treatment groups were administered with propolis extract gel. The expression of VEGF and MMP-9 was observed on days 3, 5, 7, and 9. Furthermore, mice sacrificed and the lower lip labial mucosa tissue of mice has been taken to make the histopathology anatomy preparation by means of immunohistochemical examination with monoclonal antibodies anti-VEGF and anti-MMP-9.

**Results:**

This experiment revealed higher VEGF expression and lower MMP-9 expression in the treatment group as compared to that of the control group. Analysis of Variance showed significant differences (p<0.01) of both VEGF expression and MMP-9 expression between the two groups. A Tukey’s analysis did not find strong contrasts in VEGF and MMP-9 expressions between various treatment groups. However, those between treatment and control groups were found to be considerable.

**Conclusion:**

Propolis extract gel increased the expression of VEGF and decreased that of MMP-9 during the healing process of traumatic ulcers on the oral mucosa of diabetes afflicted Wistar rats (*R. norvegicus*).

## Introduction

Ulcers represent one of the most common lesions found in the oral cavity which may be caused by stress, trauma, allergies, nutritional deficiencies, hormonal fluctuations, immunological disorders, and systemic factors. Ulcers are characterized by a loss of epithelial lining exceeding the basal membrane and affecting the lamina propria. A traumatic ulcer is one occurring in the oral cavity caused by exposure to mechanical, thermal, and chemical irritants [1,2].

Ulcers in the diabetes mellitus (DM) can be caused by protease imbalance, cytokines, and growth factors. Vascular disorders may cause tissue hypoxia leading to decreased vascular endothelial growth factor (VEGF) levels and the accumulation of advanced glycosylation end products in the tissue with chronic hyperglycemia and oxidative damage. The resulting excessive production of mitochondrial oxidative stressors leads to permanent cell damage. The VEGF plays an important role in angiogenesis. Decreased angiogenesis causes cell apoptosis and increased collagenase activities due to reduced fibroblast growth factor (FGF) and platelet-derived growth factor flow in the fibroblast synthesis process and collagen formation. The occurrence of fibroblasts and chronic reactions induces pro-inflammatory cytokines secretion (tumor necrosis factor-α and interleukin [IL]-1β). Both cytokines stimulate the synthesis of matrix metalloprotease-9 (MMP-9), causing further degradation of extracellular matrix (ECM) protein, and in turn, prolonged wound healing [3,4].

Chronic ulcers may cause pain during eating, swallowing, and talk, eventually resulting in a declining quality of life. Today, herbal and natural therapies are becoming increasingly popular. Due to their minimal side effects, bee products, including propolis, constitute the most common choice [5-7].

Propolis or bee glue is a substance produced by bees containing sticky resin and beeswax collected from plants, mainly flowers and leaf buds, mixed with bee saliva which is used to patch holes in the hive and protect the honeycomb from viruses, bacteria, and fungal attack [8]. Propolis composition is influenced by the type and age of plants, climate, and the location of source. Raw propolis consists of 50% resin, 30% wax, 10% essential oil, 5% pollen, and 5% various other organic compounds [9,10].

Propolis is an anti-inflammatory agent and contains flavonoids capable of inhibiting cyclooxygenase (COX) and lipoxygenase. Therefore, it will decrease leukotriene production and affect neutrophil phagocytosis activity suppressing, in turn, the inflammatory process. In one study of recurrent aphthous stomatitis, propolis was administered systemically at a dose of 500 mg/day for 2 weeks. The results showed evidence of a 50% decrease in the frequency of ulcer recurrence [9,11,12].

Previous laboratory research conducted by Hozzein *et al*. [13] investigated wound healing with topical application of propolis extract in rats with DM through the expression of transforming growth factor-β (TGF-β) and MMP-9. Another study by Wagh [14] incorporating application of a 3% propolis gel toothpaste demonstrated gingivitis healing due to dental plaque.

The aim of this study was to determine the effect of *Apis mellifera* propolis extract gel on VEGF and MMP-9 expression in the traumatic ulcers of rats afflicted with DM.

## Materials and Methods

### Ethical approval

This study received an ethical clearance approval letter for animal subjects from the Ethics Research Committee Faculty of Dental Medicine, Universitas Airlangga Surabaya, East Java, Indonesia, with number T024/HRECC.FODM/II/2017.

### Extract preparation

Crude propolis was harvested from a bee farm in Lawang. Wistar rats were obtained from the Biochemistry Laboratory of the Faculty of Medicine, Universitas Airlangga, Surabaya. Propolis was macerated with 70% ethanol solvent for 7 days before being subsequently filtered and evaporated by means of a vacuum rotary evaporator at 40°C to obtain viscous propolis extract [10].

The determination of a safe dose for the animal subjects was based on preliminary research. Preliminary trial of propolis extract dose with LD50 8000-40000 mg/kg for 250 g rat [13,14]. The previous study conducted by Ozan *et al*. [15] reported that a cytotoxic test of 1% propolis extract as a mouthwash showed non-toxic effects in human gingival fibroblasts. Propolis dosage of 8000-40,000 mg/kg BW for 250 g rats and dosage of 5000-10,000 mg/kg BW daily for 30 days in mice is safe and neither affects body weight nor impairs the function of liver, kidney, stomach, blood cells, or hemoglobin levels [16,17]. The extract of Lawang propolis used in this study at a concentration of 1.56% was obtained from minimal bactericidal concentration and minimal inhibitory concentration by serial propolis extract dilution method. This method adhered to that of a study by Larki-Harchegani *et al*. [18] using Lawang propolis extract which contained the highest proportion of caffeic acid (7.06%) and flavonoids (2.01%) to stimulate fibroblast cells. The preparation of propolis extract gel used 5% hydroxypropyl methylcellulose (HPMC) material as base as described earlier [19].

### Animal subjects preparations

The subjects consisted of 24 male Wistar rats (*R. norvegicus*), adapted for 7 days, provided with standard rat food constituting 10% of their body weight and drinking water at a temperature of 22°C±2°C. Wistar rats were injected intraperitoneally with a single 50 mg/kg BW dose of streptozotocin (STZ). After 3 days, rats with random blood glucose/≥200 mg/dl or fasting blood glucose ≥126 mg/dl were used as subjects in further procedures [20]. Ulceration of the labial mucosa of subjects’ lower lips was induced by thermal injury through the application of a Ball Burnisher Tip. Animal models were injected with 0.05-0.1 ml/10 g body intramuscular rodent anesthesia (ketamine, xylazine, acepromazine, and sterile isotonic saline). After 24 h, the formation of ulcers could be observed [21].

### The effect of propolis gel extract

Twenty-four male Wistar rats suffering from DM with induced ulcers on the lower labial mucosa were divided into four control groups (days 3, 5, 7, and 9) each consisting of three rats treated with 5% HPMC gel and four treatment groups (days 3, 5, 7, and 9) with the same number of rats and were administered gel propolis extract topically. Observations of VEGF and MMP-9 expression were conducted on days 3, 5, 7, and 9.

### The effect of propolis extract on VEGF and MMP-9 expression on the healing process of traumatic ulcer

The rats acclimated and the lower labial mucosa tissue was prepared for immunohistochemical imaging using monoclonal antibody (anti-VEGF) with antigen reaction (VEGF) and monoclonal antibody (anti-MMP-9) with antigen reaction (MMP-9) reacted with diaminobenzidine substrate. VGEF-expressing endothelial cells appear brown under 1000× magnification and fibroblast cells expressing MMP-9 appeared brown at 1000× magnification in 5 different fields examined by two expertise and cells were counted. The data obtained were later subjected to analysis by Analysis of Variance (ANOVA) in cases of any difference between the treatment groups, a subsequent Tukey’s test. Tukey’s analysis is a single-step multiple comparison procedure and statistical test. It can be used on raw data or in conjunction with an ANOVA (*post hoc* analysis) to find means that are significantly different from each other.

## Result

This experiment demonstrated higher VEGF expression and lower MMP-9 expression in treatment group with the application of propolis extract gel compared to the control group.

**Figure-1:**
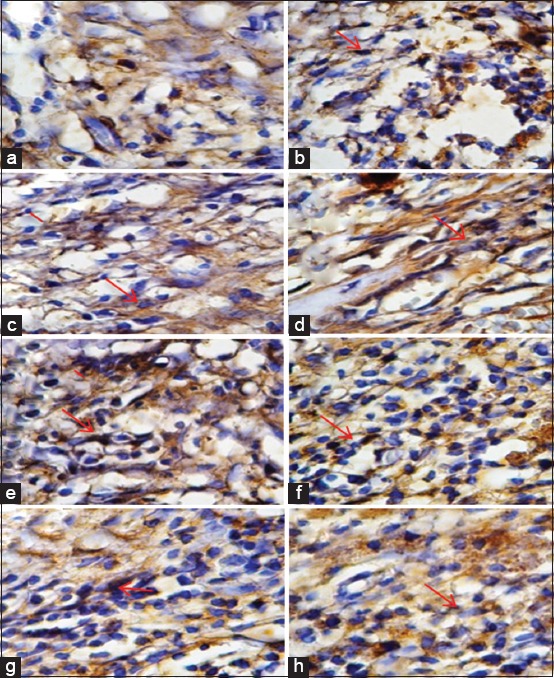
Immunohistochemistry examination showed vascular endothelial growth factor (VEGF) expression (1000×). Positive reaction showed brown color on cytoplasm indicating reaction between antigen (VEGF) and monoclonal antibody (anti-VEGF) (Red Arrow). (a) VEGF expression in endothelial cells of control group on day 3. (b) VEGF expression in endothelial cells of treatment group on day 3. (c) VEGF expression in endothelial cells of control group on day 5. (d) VEGF expression in endothelial cells of treatment group on day 5. (e) VEGF expression in endothelial cells of control group on day 7. (f) VEGF expression in endothelial cells of treatment group on day 7. (g) VEGF expression in endothelial cells of control group on day 9. (h) VEGF expression in endothelial cells of treatment group on day 9.

**Figure-2:**
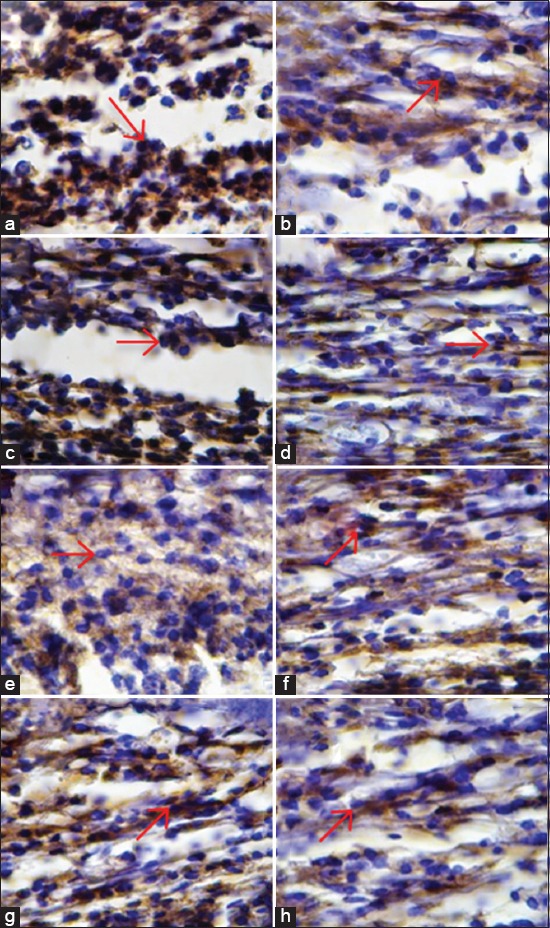
Immunohistochemistry examination showed matrix metalloproteinase-9 (MMP-9) expression (1000×). Positive reaction showed brown color on cytoplasm indicated reaction between antigen (MMP-9) and monoclonal antibody (anti-MMP-9) (Red Arrow). (a) MMP-9 expression in endothelial cells of control group on day 3. (b) MMP-9 expression in endothelial cells of treatment group on day 3. (c) MMP-9 expression in endothelial cells of control group on day 5. (d) MMP-9 expression in endothelial cells of treatment group on day 5. (e) MMP-9 expression in endothelial cells of control group on day 7. (f) MMP-9 expression in endothelial cells of treatment group on day 7. (g) MMP-9 expression in endothelial cells of control group on day 9. (h) MMP-9 expression in endothelial cells of treatment group on day 9.

ANOVA analysis showed significant differences (p<0.01) in VEGF expression and MMP-9 expression between the groups (Tables-1 and 2). There were also significant differences in the mean VEGF and MMP-9 expression (p<0.01) that can be seen in Figure-1. Tukey’s analysis did not identify significant differences in VEGF and MMP-9 expression between the treatment groups, while the differences between the control groups were found to be significant. Immunohistochemistry examination showed that VEGF expression and MMP-9 have significant difference between treatment and control groups that can be seen in Figure-2.

**Table-1:** Description of mean±SD and statistical differences of VEGF expression between control and treatment groups with propolis extract and HPMC 5% application on days 3, 5, 7, and 9.

Time	VEGF expression control group	VEGF expression treatment group	Oneway ANOVA p
Day 3	5.7±1.2	13.0±2.0	0.000*
Day 5	3.7±1.2	15.0±1.0	0.000*
Day 7	3.0±1.0	11.7±2.1	0.000*
Day 9	7.0±2.0	14.3±1.5	0.000*

* Significant p>0.01. SD=Standard deviation, VEGF=Vascular endothelial growth factor, HPMC: Hydroxypropyl methylcellulose

**Table-2:** Description of mean±SD and statistical differences of MMP-9 expression between control and treatment groups with propolis extract and HPMC 5% application on days 3, 5, 7, and 9.

Time	MMP-9 expression control group	MMP-9 expression treatment group	Oneway ANOVA p
Day 3	7.3±2.1	8.0±1.0	0.000*
Day 5	9.7±1.2	7.0±2.6	0.000*
Day 7	11.3±1.5	7.3±1.5	0.000*
Day 9	15.7±1.5	4.0±1.0	0.000*

* Significant p>0.01. SD: Standard deviation, MMP-9: Matrix metalloproteinase9, HPMC: Hydroxypropyl methylcellulose

## Discussion

This research constituted an experimental investigation with post-test only control group design. The research samples consisted of white, male, 2-3 month-old, Wistar rats (*R. norvegicus* strain Wistar), with a body weight of 200-250 g, chosen as animal models due to their sharing the same metabolism as humans. The results can, therefore, be generalized to humans. Moreover, rats are easy to handle and unaffected by the hormonal system, making them homogeneous as research subjects [22,23].

50 mg/kg BW STZ was injected intraperitoneally into each Wistar rat to induce a DM condition [20,22]. On day 3, rats were diagnosed with DM if, and after administration of STZ, blood glucose levels had been raised to ≥200 mg/dl [20,24].

Ulcers were induced using a burnisher heated for 60 s which was then applied without pressure to the lower labial mucosa of the rat for 1 s so as to prevent endothelial layer perforation. Clinical features of ulcers appeared after 24 h. This thermal ulcer induction method was based on research by Subramanian *et al*. [21].

HPMC 5% was used as a thickening agent and stabilizer in gel preparation due to its supple yet solid consistency, ease of application to the ulcer, durability and lack of influence on base substance function, none of which affected the research results. Gel was used as a covering agent since it is biocompatible, biodegradable, and hemostatic [19].

The treatment group demonstrated more rapid clinical healing than that of the control group. In the treatment group, erythema appeared on the day 5, while in the day 7, the mucosa assumed a reddish-white appearance, before being restored to normal mucosa on the day 9. These findings were similar to those of research conducted by Günay *et al*. [25], which proved the role of flavonoids and Caffeic Acid Phenylethyl Ester (CAPE) as antioxidants and anti-inflammatories that increased reepithelization and accelerated the post-extraction healing process in sockets.

CAPE plays a role in suppressing T-cell activity. CAPE inhibits nuclear transcription factor kappa B (NF-kB) and IL-2 stimulants that promote T-cell proliferation. CAPE also plays an anti-inflammatory role in inhibiting arachidonic acid secretion in cell membranes, the production of prostaglandin E2, and histamine release leading to the suppression of COX-1 and COX-2 activation and the inhibition of gene expression from COX-2. Inhibition of lipoxygenase and the COX pathway by CAPE will reduce vasodilation and blood flow, eventually decreasing the migration of leukocytes polymorphonuclear leukocyte (PMN) to the inflamed area [3,11].

VEGF will trigger cell proliferation and differentiation in angiogenesis and increase endothelial cell proliferation, differentiation, and migration [26,27]. MMP serves to degrade ECM in the inflammatory phase, degrade tissue around the site of new blood vessels, and play a role in the contraction and remodeling of tissue during the remodeling phase. In chronic wounds, an increase in MMP-9 may occur [28,29].

On the days 3 and 5, the control groups clinically presented yellowish ulcers. On the day 7, the ulcer began to shrink with necrotic tissue, while on the 9^th^ day, it had healed as evidenced by the presence of scar tissue. In conditions of DM, the healing process was inhibited since microangiopathy caused tissue hypoxia, increased inflammatory mediators, decreased leukocytes, angiogenesis, vasculogenesis, proliferation, and fibroblast migration, increased apoptosis of fibroblasts, and reduced collagen formation [6,17,30-32]. Hence, the control group experienced a more prolonged clinical healing process accompanied by cicatrix formation. This was consistent with Brizeno *et al*. study [33] that untreated ulcers in DM and chronic inflammatory conditions showed decreased collagen synthesis, thus resulting in slower healing.

The results of a statistical and mean test showed significant differences (p<0.000) of VEGF expression in each treatment group and control group. The expression in the treatment group was higher than that of the control group on days 3, 5, 7, and 9. There was also an increase in VEGF expression from day 3 to day 5 and day 7 to day 9. This demonstrated the role of flavonoids and propolis in stimulating macrophages and PMN. Macrophages release VEGF and FGF-2 to induce angiogenesis which represented a critical point between chronic inflammation and fibrosis. Stimulation of angiogenesis results in migration, proliferation, and differentiation of endothelial cells. Capillary proliferation acted as a pathway for oxygenation and micronutrients for tissue growth [27,28,31]. Previous research had examined wound healing in DM mice with topical application of propolis extract and showed an increased expression of TGF-β and MMP-9 [13].

A statistical test of MMP-9 expression revealed that there were no significant differences within the treatment group; however, significant differences did exist within the control group. This proved that in the treatment group, propolis extract containing CAPE (phenolic acids like CAPE) and bioactive substances promoted anti-inflammatory activity that inhibited phospholipase in arachidonic acid cascade, thereby preventing the release of prostaglandins and leukotrienes. CAPE has lipophilic properties which allow penetration into cells, inhibiting the release of pro-inflammatory cytokines and increasing the anti-inflammatory cytokines simultaneously. CAPE possesses analgesic activities that induce macrophages, increase growth factors, inhibit MMP-9, and promote ECM remodeling [3,8,33]. Moreover, several studies have shown that topical application of propolis in post-tooth extraction reduces inflammation since CAPE mechanisms were able to inhibit NF-kB and arachidonic acid cascade [28,32-34].

## Conclusion

From the results of this study, it can be concluded that topical propolis gel extract could increase VEGF expression and decrease MMP-9, thus indicating the presence of angiogenesis, decrease collagen degradation, therefore accelerating the wound healing process within ulcers in DM afflicted rat model.

## Authors’ Contributions

DSE: Conception and design of the study; AP: Acquisition of data; AP and DSE: Analysis and/or interpretation of the data; DSE: Drafting and revising the manuscript critically for important intellectual content. All authors read and approved the final manuscript.
